# Gamma Interferon Alters Junctional Integrity via Rho Kinase, Resulting in Blood-Brain Barrier Leakage in Experimental Viral Encephalitis

**DOI:** 10.1128/mBio.01675-19

**Published:** 2019-08-06

**Authors:** Stephanie Bonney, Scott Seitz, Caitlin A. Ryan, Kenneth L. Jones, Penny Clarke, Kenneth L. Tyler, Julie A. Siegenthaler

**Affiliations:** a Department of Pediatrics, Section of Developmental Biology, University of Colorado, School of Medicine, Aurora, Colorado, USA; b Cell Biology, Stem Cells, and Development Graduate Program, University of Colorado, School of Medicine, Aurora, Colorado, USA; c Microbiology Graduate Program, University of Colorado, School of Medicine, Aurora, Colorado, USA; d Department of Neurology, University of Colorado, School of Medicine, Aurora, Colorado, USA; e Department of Pediatrics, Section of Hematology, Oncology, and Bone Marrow Transplant, University of Colorado, School of Medicine, Aurora, Colorado, USA; Baylor College of Medicine; National Institutes of Health; University of Utah School of Medicine

**Keywords:** blood-brain barrier, encephalitis, viral encephalitis, interferon gamma, reovirus

## Abstract

Blood-brain barrier (BBB) breakdown is a hallmark of many diseases of the central nervous system (CNS). Loss of BBB integrity in CNS diseases such as viral encephalitis results in the loss of nutrient/oxygen delivery, rapid infiltration of immune cells, and brain swelling that can exacerbate neuronal injury. Despite this, the cellular and molecular mechanisms that underlie BBB breakdown in viral encephalitis are incompletely understood. We undertook a comprehensive analysis of the cellular and molecular signaling events that induce BBB breakdown in an experimental model of virus-induced encephalitis in which neonatal mice are infected with reovirus (serotype 3 strain Abney). We show that BBB leakage during reovirus infection correlates with morphological changes in the vasculature, reductions in pericytes (BBB supporting cells), and disorganization of vascular junctions. Pathway analysis on RNA sequencing from brain endothelial cells identified the activation of interferon (IFN) signaling within the brain vasculature following reovirus infection. Our *in vitro* and *in vivo* studies show that type II IFN mediated by IFN-γ, a well known antiviral signal, is a major contributor to BBB leakage during reovirus infection. We show that IFN-γ reduces barrier properties in cultured brain endothelial cells through Rho kinase (ROCK)-mediated cytoskeletal contractions, resulting in junctional disorganization and cell-cell separations. *In vivo* neutralization of IFN-γ during reovirus infection significantly improved BBB integrity, pericyte coverage, attenuated vascular ROCK activity, and junctional disorganization. Our work supports a model in which IFN-γ acts directly on the brain endothelium to induce BBB breakdown through a mechanism involving ROCK-induced junctional disorganization.

## Abstract (precis)

In an experimental viral encephalitis mouse model in which mice are infected with reovirus, we show that IFN-γ induces blood-brain barrier leakage. We show that IFN-γ promotes Rho kinase activity, resulting in actin cytoskeletal contractions in the brain endothelium that lead to vascular junctional disorganization and cell-cell separations. These studies now provide insight into a previously unknown mechanism for how blood-brain barrier breakdown occurs in viral encephalitis and implicates IFN-γ-Rho kinase activity as major contributor to this phenomenon. By identifying this mechanism of blood-brain barrier breakdown, we now provide potential therapeutic targets in treating patients with viral causes of encephalitis with the hope of limiting damage to the central nervous system.

## INTRODUCTION

Viral encephalitis is an acute inflammatory process caused by viral infections within the central nervous system (CNS). It is a disease that is often associated with blood-brain barrier (BBB) leakage which can enhance neuronal damage, the rates of immune invasion, and swelling of the CNS (1–3). Thus, it is important to understand the molecular and cellular events that result in BBB breakdown during viral encephalitis.

Endothelial cells make up the brain vasculature and obtain BBB properties to limit extravasation of harmful substances and immune cells into the CNS. Brain endothelial cells are uniquely enriched with tight junctions (TJs) sealing the endothelial membranes, and together with adherens junctions (AJs), form a continuous nearly impermeable vascular network. The actin cytoskeleton supports junctional integrity by reinforcing endothelial cell shape, permitting strong contacts with neighboring endothelial cells (2, 4–6). These and other BBB properties are crucially supported by pericytes, a type of mural cell (7, 8). Importantly, these barrier properties (TJ enrichment and brain pericyte coverage) and function (exclusion to small molecular tracers) are present at birth (9, 10).

TJs have been the main focus for understanding BBB leakage in viral encephalitis. Specifically, the TJ proteins claudin-5, occludin, and ZO-1 are reportedly reduced, correlating with BBB leakage in mouse models of viral encephalitis (11–14). In contrast, other studies have shown that TJ protein expression is unaltered but that localization at the cell borders appears to be disrupted during infection or inflammatory instances (15–17). The latter studies have implicated the actin cytoskeleton in regulating TJ stability and localization at cell junctions during viral infection. Daniels et al. found that interferon alpha receptor (IFNAR) signaling prevents barrier disruption by activating Rac1, a GTPase that is responsible for stabilizing the endothelial actin cytoskeleton (15). These studies suggest that type I interferon (IFN) signaling, mediated by IFNAR, is barrier protective during CNS viral infection. Interestingly, recent studies implicate type II IFN signaling, mediated by IFN-γ, as being barrier disruptive during viral encephalitis. (11, 13). However, these studies did not identify the underlying mechanism of how IFN-γ directly reduces barrier properties in brain endothelial cells. In addition, a majority of these studies have focused solely on TJs; therefore, the effect of CNS viral infection on other brain vascular properties, such as AJs and pericytes, is unknown.

We sought to reveal the mechanisms of BBB breakdown during encephalitis by utilizing a classical mouse model of viral encephalitis in which perinatal mice are infected with reovirus serotype 3 strain Abney (T3A), a clinically isolated neurotropic reovirus strain. Human reovirus infection has been associated with meningitis and encephalitis in infants and children, although it more typically results in mild upper respiratory or gastrointestinal disease (18, 19). Nonetheless, reovirus-induced encephalitis mouse models have been very informative for understanding the pathogenesis of viral encephalitis (20, 21). Using the reovirus-induced encephalitis mouse model, we found that BBB leakage is coincident with morphological changes in the brain vasculature, loss of pericytes, and disorganization of vascular junctions. Transcriptome analysis of brain endothelial cells from reovirus infected mice identified activation of IFN signaling in the brain vasculature. Neutralization of IFN-γ attenuated BBB leakage during reovirus infection. Our *in vitro* and *in vivo* studies found that IFN-γ directly disrupts the localization of AJs and TJs proteins in brain endothelial cells. We show this effect is through IFN-γ-Rho kinase-mediated actin cytoskeletal contractions and cell-cell separations ultimately contributing to barrier leakage. Our studies provide essential insight into the previously unknown mechanisms of IFN-γ-mediated BBB disruption during viral encephalitis.

## RESULTS

### Blood-brain barrier breakdown occurs at late stages of CNS reovirus infection.

**FIG 1:**
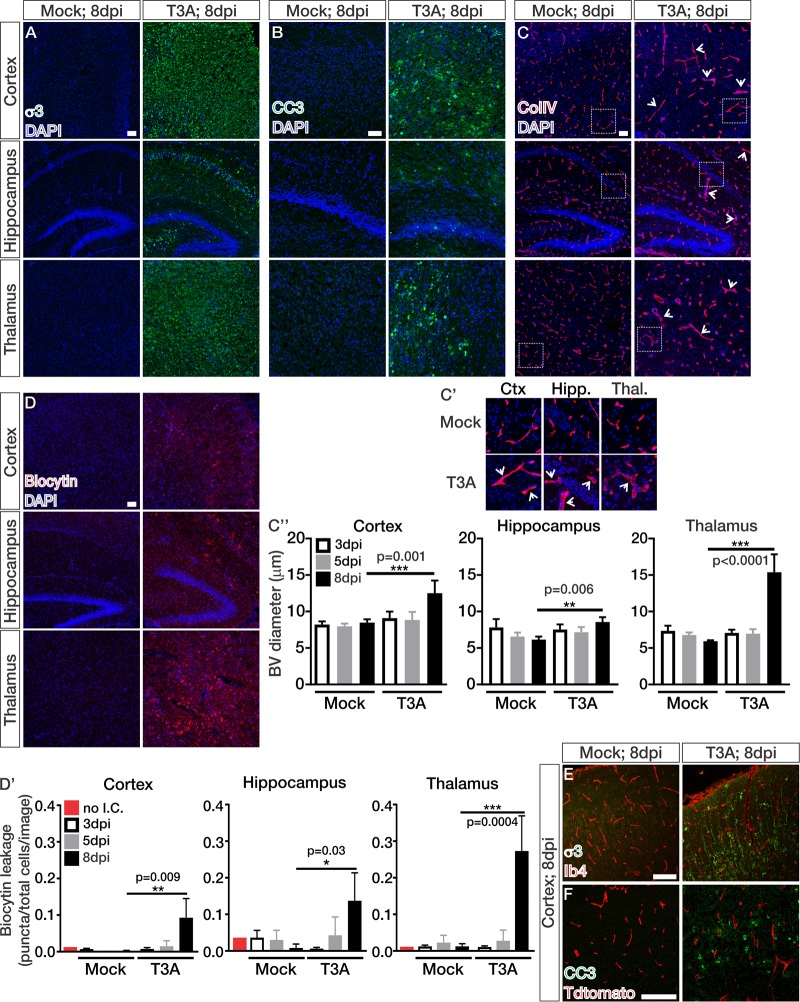
Blood-brain barrier breakdown occurs at late stages of CNS reovirus infection. (A to D) Immunohistochemical (IHC) images from the cortices, hippocampi, and thalami of mock- and T3A-infected mice at 8 dpi immunostained for the T3A viral protein σ3 (green) (A), cleaved caspase 3 (CC3; green) (B), collagen-IV (ColIV; red) (C), and Biocytin-TMR (red) with DAPI (blue) (D). (C′) Enlarged images to depict vessel hyperplasia (arrows) after 8 days of T3A infection. (C″ and D′) Quantification of the blood vessel diameter (μm; *n* = 4 animals/region/time point) (C″) or Biocytin-TMR leakage (D′) using threshold analysis (Biocytin puncta/total cells/image; *n* = 3 animals/region/time point) in mock- and T3A-infected mice at 3 (white), 5 (gray), or 8 dpi (black) shows significant increases in blood vessel diameter and Biocytin-TMR leakage 8 days after T3A infection in the cortex, hippocampus, and thalamus. The red bar in panel D′ indicates Biocytin-TMR-streptavidin background levels in a P7 pup that did not receive an intracranial injection (no I.C.) of PBS or T3A. (E and F) IHC images from the cortices of mock- and T3A-infected mice at 8 dpi immunostained for σ3 (green) and isolectin-b4 (Ib4; red) (E) or CC3 (green) and Tdtomato expression (red) (F) in *Cdh5^creERT2/+^*; *Ai14^fl/+^* mice. Scale bars, 50 μm.

Our first aim was to uncover if and when BBB leakage occurs in a classic experimental mouse model of viral encephalitis. In this model, 2-day-old pups are intracranially inoculated with the T3A strain of reovirus or phosphate-buffered saline (PBS) for mock controls. Perinatal mice intracranially infected with reoviruses generally succumb to infection at 9 or 10 days postinfection (dpi) (22). To detect BBB breakdown throughout T3A infection, we analyzed brain tissue from mock- and T3A-infected mice at early (3 dpi), middle (5 dpi), and late (8 dpi) stages of disease. Primary inoculation with T3A or PBS was performed intracranially along the midline of the cortex of either Swiss Webster or *Cdh5cre^ERT2/+^*; *Ai14^fl/+^* pups with Tdtomato expression in the vasculature. Virus then spreads to secondary sites such as the hippocampus and thalamus (22). By 8 dpi, a substantial number of T3A (reoviral protein σ3)-infected cells were observed in the cortex, hippocampus, and thalamus (Fig. 1A). This stage of disease is associated with extensive cell death, as indicated by labeling with cleaved caspase 3 (CC3), a marker of apoptosis, in these brain regions (Fig. 1B) (21, 22). At this time point, we also observed significant increases in blood vessel diameter (arrows; Fig. 1C to C″), an indication of vascular hyperplasia (23). To measure BBB integrity throughout T3A infection, we administered intraperitoneal injections of Biocytin-TMR, an 870-Da fluorescent tracer that tests paracellular/junctional integrity and is normally restricted from entering the CNS by an intact BBB (24). Eight days after T3A infection, we observed significant leakage of Biocytin-TMR into the cortex, hippocampus, and thalamus, suggesting BBB dysfunction occurs at later stages of disease (Fig. 1D and D′). Intracranial injections alone did not cause BBB leakage (red; Fig. 1D′). Importantly, T3A did not directly infect the vasculature (Fig. 1E), and cell death was not detected in the blood vessels (Fig. 1F), ruling out the possibility that viral infection or cell death in the endothelium contributes to BBB breakdown. Together, these data show that BBB breakdown occurs at late stages of disease that coincide with significant T3A infection and neural cell death.

### Pericyte loss and junctional disorganization occur at late stages of CNS reovirus infection.

**FIG 2:**
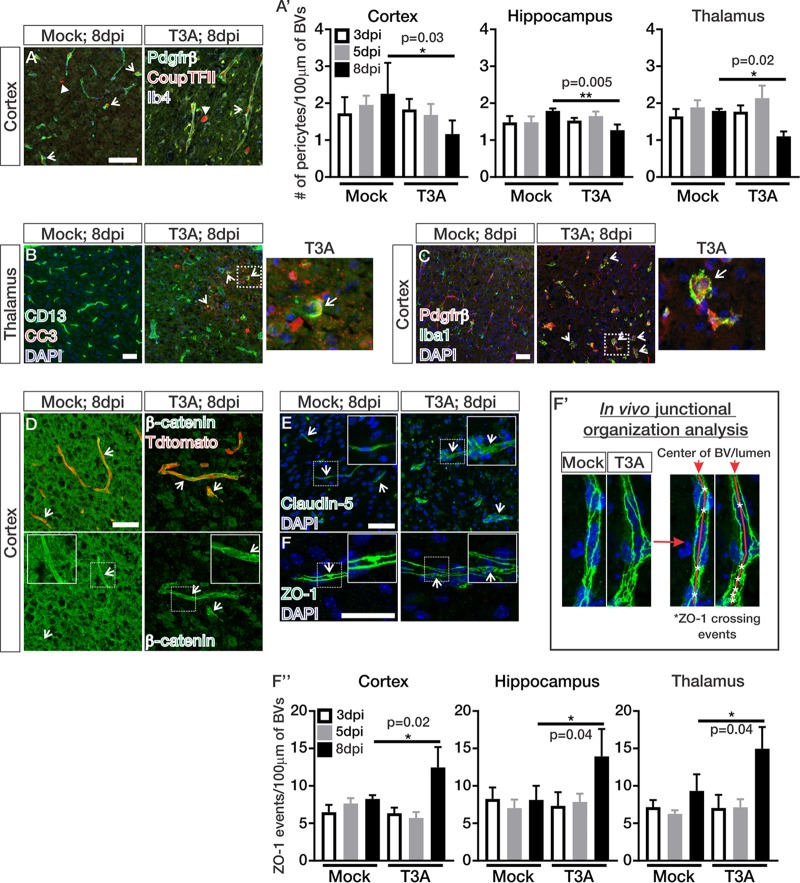
Pericyte loss and junctional disorganization occur at late stages of CNS reovirus infection. (A to C) IHC images of mock- and T3A-infected mice at 8 dpi immunostained for Pdgfrβ (green)- and CoupTFII (red)-expressing pericytes (arrows) around Ib4^+^ blood vessels (BVs; blue) in the cortex (in panel A. CoupTFII-expressing interneurons are indicated by closed arrows. (B and C) CD13-labeled pericytes (green), cleaved caspase-3 (CC3; red), and Ib4^+^ or Tdtomato labeled BVs (blue) (arrows indicate apoptotic pericytes in the thalamus) (B) and Pdgfrβ (red), Iba1 (green), and DAPI (blue) (arrows indicate phagocytosed Pdgfrβ^+^ fragments in Iba1^+^ phagocytes in the cortex) (C). (A′) Quantification of Pdgfrβ/CoupTFII^+^ pericytes/100 μm of BVs in mock- and T3A-infected (*n* = 4 animals/region/time point) mice at 3 (white), 5 (gray), or 8 dpi (black). Analysis indicated significant decreases in pericytes/100 μm of BV length 8 days after T3A infection in the cortex, hippocampus, and thalamus. (D to F) IHC images from the cortices of mock- and T3A-infected mice at 8 dpi immunostained for the adherens junction [AJ] adaptor protein β-catenin (green; arrows) and Tdtomato (red) expression in the vasculature (D), claudin-5 (tight-junction [TJ] protein; green; arrows) and DAPI (blue) (E), and ZO-1 (AJ and TJ adaptor proteins; green; arrows) and DAPI (blue) (F). The arrows show intact junctions across the endothelium. (F′) Junctional analysis method for counting the number of ZO-1 crossing events (asterisks) down the center of the longitudinal BVs (red line). (F″) Quantification of ZO-1 organization in mock- and T3A-infected mice at 3 (white), 5 (gray), or 8 dpi (black) (*n* = 3 animals/region/time point; as shown in panel F′). Analysis shows significant increases in ZO-1 crossing events/100 μm of BV length at 8 days after T3A infection in the cortex, hippocampus, and thalamus, indicating junctional disorganization. Scale bars, 50 μm.

BBB leakage and morphological alterations in the brain vasculature during T3A infection prompted us to next assess cellular properties of the BBB. We began by assessing whether the pericyte population is affected by T3A infection. By performing immunohistochemical (IHC) analyses for the pericyte markers Pdgfrβ and CoupTFII, we identified pericytes by their perivascular location on Ib4^+^ capillaries and coexpression of Pdgfrβ (membrane) and CoupTFII (nuclear). We found significant reductions in the number of pericytes at late stages of T3A infection (8 dpi; Fig. 2A and A′). Occasional CD13-labeled pericytes were CC3 positive at 8 dpi (arrows; Fig. 2B), indicating pericytes undergo apoptosis at late stages of infection. Interestingly, we also observed engulfment of Pdgfrβ cells and fragments by Iba^+^ phagocytes at late stages of T3A infection (arrows; Fig. 2C). These data indicate that the loss in pericytes during CNS reovirus infection may be due to apoptosis and/or engulfment by phagocytic cells. Considering the key role of pericytes in BBB integrity (7, 8), pericyte loss could contribute to BBB leakage during T3A infection.

Leakage of Biocytin-TMR is consistent with breakdown of the paracellular barrier that is normally maintained by TJs and AJs between adjacent endothelial cells (2, 24). Therefore, we next investigated the expression and organization of AJ and TJ proteins in the brain vasculature throughout T3A infection by using an IHC approach. The level of β-catenin (AJ adaptor protein; Fig. 2D) and claudin-5 (TJ protein; Fig. 2E) protein expression appeared normal however their junctional localization appeared disorganized at late stages of infection (arrows; Fig. 2D and E). The junctional disorganization was more apparent in ZO-1 labeling of blood vessels, an AJ and TJ adaptor protein (25, 26), which revealed potential disorganization in junctional zones 8 days after T3A infection (Fig. 2F). The highly specific labeling of endothelial junctions by ZO-1 permitted us to assess how junctional organization is affected during T3A infection. To quantify junctional organization in the vasculature throughout T3A infection, we devised a strategy to quantify the number of ZO-1 crossing events (indicated by asterisks) down the center of blood vessels (red line) and normalized that to 100 μm of blood vessel length (Fig. 2F′). Blood vessels in mock-infected animals had ZO-1 crossing events that appeared evenly spaced out with an average of six to seven ZO-1 crossings per 100 μm of blood vessel length (Fig. 2F′ and F″). ZO-1 crossing events in blood vessels of T3A-infected animals at 8 dpi appeared to be inconsistently spaced, and the numbers of ZO-1 crossings per 100 μm of blood vessel length were significantly increased in the cortex, hippocampus, and thalamus (Fig. 2F′ and F″). Together, these data suggest that junctional organization is greatly altered at late stages of T3A infection, likely affecting BBB integrity and causing leakage.

### IFN signaling is active in the brain vasculature during CNS reovirus infection.

**FIG 3:**
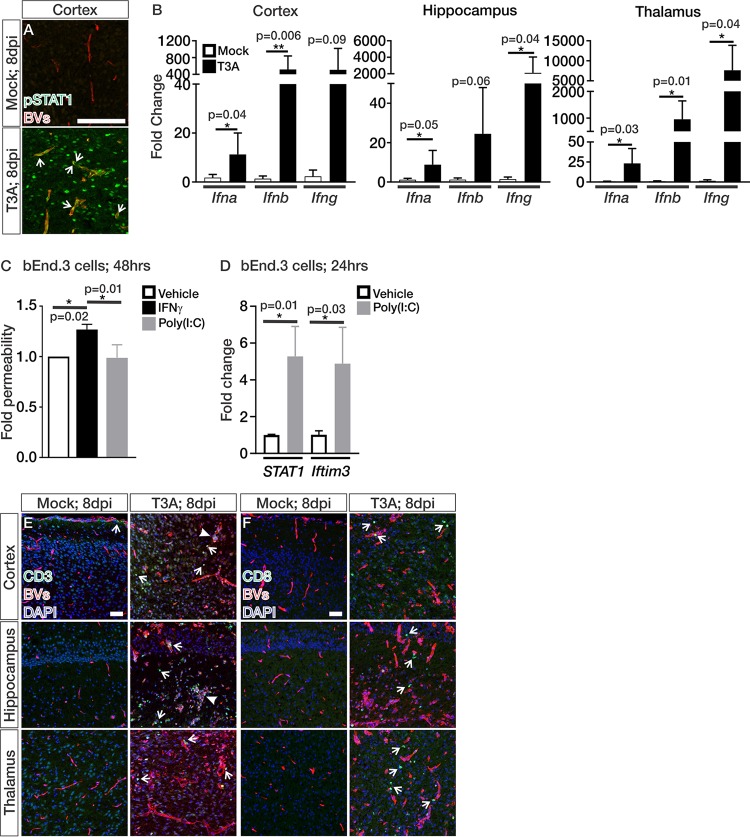
IFN signaling is active in the brain endothelium during CNS reovirus infection. (A) IHC images from the cortex of mock- and T3A-infected *Cdh5cre^ERT2/+^*; *Ai14^fl/+^* or Swiss Webster mice at 8 dpi immunostained for pSTAT1 (green) and Ib4^+^ or Tdtomato expression (BVs; red). Arrows indicate pSTAT1^+^ brain endothelial cells. (B) Transcript expression of *Ifna*, *Ifnb*, and *Ifng* in the cortices, hippocampi, and thalami of mock-infected (white; *n* = 5) and T3A-infected (black; *n* = 6) mice at 8 dpi. (C) Fold changes in permeability of bEnd.3 cultures (*n* = 3 independent experiments; after 60 min of NaF leakage) after 48 h of treatment with vehicle (white), IFN-γ (black), or poly(I:C) (gray) indicate that IFN-γ specifically reduces barrier properties in brain endothelial cells. (D) Fold changes in *STAT1* and *Ifitm3* transcript expression in bEnd.3 cultures (*n* = 3) after 24 h of treatment with vehicle (white) or poly(I:C) (gray). (E and F) IHC images from the cortices, hippocampi, and thalami of *Ai14^fl/+^* or Swiss Webster mice at 8 dpi immunostained for CD3 (green) (E) or CD8 (green) (F) with Ib4^+^ or Tdtomato expression (BVs; red) and DAPI (blue). Arrows indicate CD3^+^ and CD8^+^ T cells in the neural parenchyma and around the blood vessels. Closed arrows indicate regions with a high presence of T cells and potential perivascular cuffing. Scale bars, 50 μm.

To identify potential pathways that may be initiating these changes in the vasculature during T3A infection, we performed RNA sequencing (RNA-seq) and pathway analysis on Tdtomato^+^ fluorescence-activated cell-sorted brain endothelial cells from *Cdh5^creERT2/+^*; *Ai14^fl/+^* mock- and T3A-infected animals at 6 dpi, prior to when we detect BBB leakage. *Cdh5^creERT2/+^* recombination is specific to the blood vasculature (27), and we observed low expression of neuronal (NeuN/*Rbfox3 *<* *5 fragments per kilobase million [FPKM]), oligodendrocyte (*Olig2* ∼8 FPKM), and microglial (*Cxcr3 *<2 FPKM) markers. However, we did observe some expression of the astrocyte-specific marker, *S100b* (∼40 FPKM). This is much lower than the expression of brain endothelial enriched genes (*Cldn5* ∼3,000 FPKM, *Slc2a1* ∼900 FPKM), demonstrating that the vast majority of cells captured for RNA-seq were brain endothelial cells. Ingenuity Pathway Analysis on this transcriptome data identified significant activation of IFN signaling in the brain endothelial cells of T3A-infected mice (*P* = 1.75E–12). We verified active IFN signaling in the endothelium by the presence of numerous pSTAT1^+^ endothelial cells at 8 days after T3A infection (arrows; Fig. 3A). Furthermore, activation of endothelial-IFN signaling, as indicated by pSTAT1, correlated with the timing of BBB leakage at 8 dpi (Fig. 1D) and upregulation of IFN-α, IFN-β, and IFN-γ transcripts in the cortices, hippocampi, and thalami of T3A-infected mice (8 dpi; Fig. 3B). To understand whether type I or II IFN signaling is capable of disrupting barrier properties in brain endothelial cells, we performed permeability assays and found that IFN-γ induced leakage of sodium fluorescein (NaF) in monolayers of bEnd.3 cells, a mouse brain endothelioma cell line (Fig. 3C). The type I IFN activator poly(I:C) significantly induced the expression of the IFN targets *STAT1* and *Ifitm3* (Fig. 3D) but did not alter barrier properties in bEnd.3 cells (Fig. 3C). Together, these data show that IFN signaling is active in the brain endothelium during CNS infection and that IFN-γ, but not type I IFN, signaling reduces barrier properties in cultured brain endothelial cells.

To identify the potential cellular source of IFN-γ, we performed IHC analyses for the presence of T cells, well-known producers of IFN-γ (3). We detected a substantial amount of CD3^+^ and CD8^+^ T cells in the neural parenchyma and attached along the endothelium at late stages of disease in the cortex, hippocampus, and thalamus (open arrows; Fig. 3E and F). There were some instances of concentrated CD3^+^ T cells (closed arrows; Fig. 3E), indicative of perivascular cuffing; however, not all vessels had this feature. These data suggest that T cells are a likely source of IFN-γ that activates endothelial-IFN signaling at the late stages of reovirus infection. Of note, we occasionally found CD3^+^ T cells in the meninges of mock-infected animals (arrows; Fig. 3E).

### Neutralization of IFN-γ attenuates BBB leakage and some disease progression during CNS reovirus infection.

**FIG 4:**
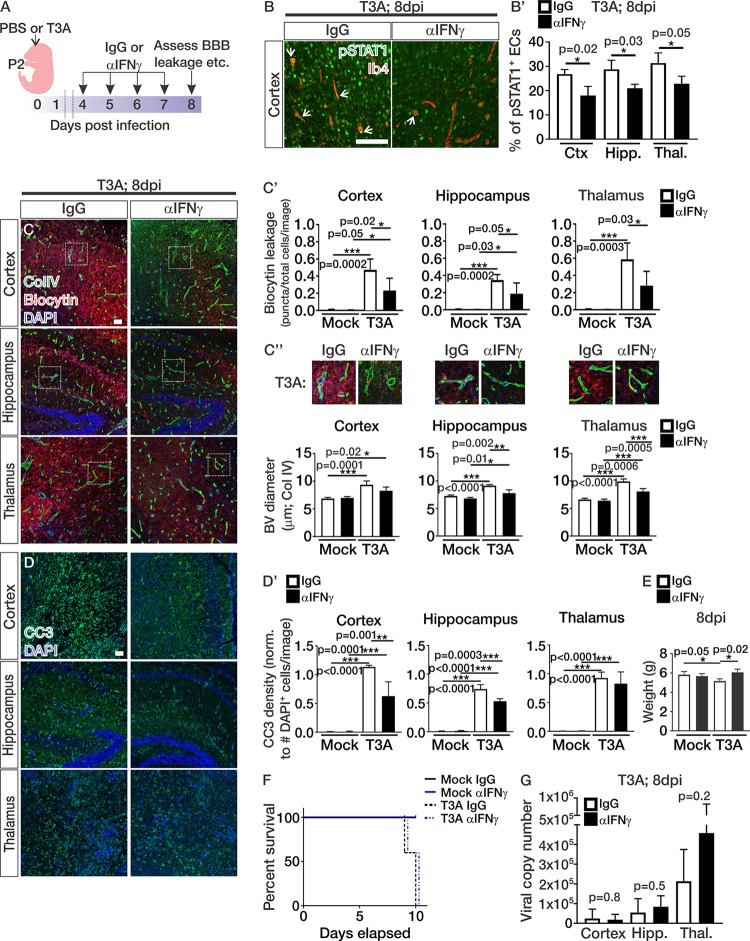
Neutralization of IFN-γ during CNS reovirus infection attenuates BBB leakage and some disease progression. (A) Experimental design for pups infected 2 days after birth with PBS (mock) or reovirus T3A and treated with IgG or αIFN-γ from 4 to 7 dpi, followed by analysis of the BBB breakdown, vascular morphology, and BBB properties at 8 dpi. (B to D) Immunohistochemical images from the cortices, hippocampi, and thalami of mock- and T3A-infected mice treated with IgG or αIFN-γ showing pSTAT1 (green) and Ib4 (red) (B), Biocytin-TMR (red) and the blood vessel marker collagen-IV (ColIV; green) (C), and cleaved caspase 3 (CC3; green) with DAPI (blue) (D) at 8 dpi. Scale bars, 50 μm. (B′) Quantification of the percentages of pSTAT1^+^ endothelial cells in T3A-infected mice treated with IgG (white; *n* = 3) and αIFN-γ (black; *n* = 3). (C′) Biocytin-TMR leakage using threshold analysis (Biocytin puncta/total cells/image) in the cortices, hippocampi, and thalami of mock-infected (*n* = 5 animals/treatment) and T3A-infected (*n* = 5 animals/treatment) mice treated with IgG (white) or αIFN-γ (black). (C″) Enlarged images of ColIV-labeled vessels (green) and Biocytin-TMR leakage (red) with quantification of blood vessel diameter (μm) in the cortices, hippocampi, and thalami of mock-infected (*n* = 4 animals/treatment) and T3A-infected (*n* = 3 animals/treatment) mice treated with IgG (white) or αIFN-γ (black). (D′) Quantification of CC3 density using threshold analysis (CC3^+^ cells/total cells/image) in the cortices, hippocampi, and thalami of mock-infected (*n* = 4 animals/treatment) and T3A-infected (*n* = 3 animals/treatment) mice treated with IgG (white) or αIFN-γ (black). (E) Body weights of mock-infected (*n* = 4 animals/treatment) and T3A-infected (*n* = 3 animals/treatment) mice treated with IgG (white) or αIFN-γ (black) at 8 dpi. (F) The percent survival in mock-infected (solid lines; *n* = 10 animals/treatment) and T3A-infected (dashed lines; *n* = 10 animals/treatment) mice after IgG (black) or αIFN-γ (blue) treatment shows that there is no effect on survival in mice treated with αIFN-γ. (G) Viral copy numbers of reovirus T3A in the cortices, hippocampi, and thalami of T3A-infected mice treated with IgG (white; *n* = 5) and αIFN-γ (black; *n* = 6).

To test whether IFN-γ plays a role in BBB leakage during reovirus infection, we treated pups with a monoclonal neutralizing IFN-γ antibody (αIFN-γ) from 4 to 7 dpi and analyzed Biocytin-TMR leakage and vascular morphology (Fig. 4A). Neutralization of IFN-γ in these experiments reduced the percentage of pSTAT1-positive endothelial cells, verifying that the αIFN-γ treatment is effective for blocking endothelial-IFN signaling (Fig. 4B and B′). αIFN-γ treatment during T3A infection significantly reduced Biocytin-TMR leakage in the cortex, hippocampus, and thalamus (Fig. 4C and C′), similar to what is observed in other viral encephalitis mouse models (11, 13). T3A-infected mice treated with IgG and αIFN-γ displayed significant increases in blood vessel diameters in the cortex, hippocampus, and thalamus compared to mock-infected animals treated with IgG and αIFN-γ (Fig. 4C and C″). However, neutralization of IFN-γ significantly attenuated the dilation in the vasculature in comparison to T3A IgG-treated mice (Fig. 4C and C″). Together, these data demonstrate that blocking IFN-γ during T3A infection attenuates BBB leakage and morphological changes within the vasculature.

The improvement in BBB leakage by neutralizing IFN-γ during reovirus infection prompted us to assess other disease parameters, specifically neuronal cell death and animal survival. Quantification of cell death using the presence of CC3 showed attenuation of cell death in the cortex and hippocampus of T3A-infected mice treated with αIFN-γ (Fig. 4D and D′). We did observe improvements in animal weight (Fig. 4E); however, the αIFN-γ treatments did not alter animal survival (Fig. 4F). The viral copy numbers were similar between IgG- and αIFN-γ-treated animals (Fig. 4G), indicating that blockage of IFN-γ does not heighten viral replication. Although treatment with αIFN-γ during T3A infection improves animal weight and partially reduces neuronal cell death, the treatments do not significantly attenuate disease progression.

### Neutralization of IFN-γ attenuates pericyte loss during CNS reovirus infection.

**FIG 5:**
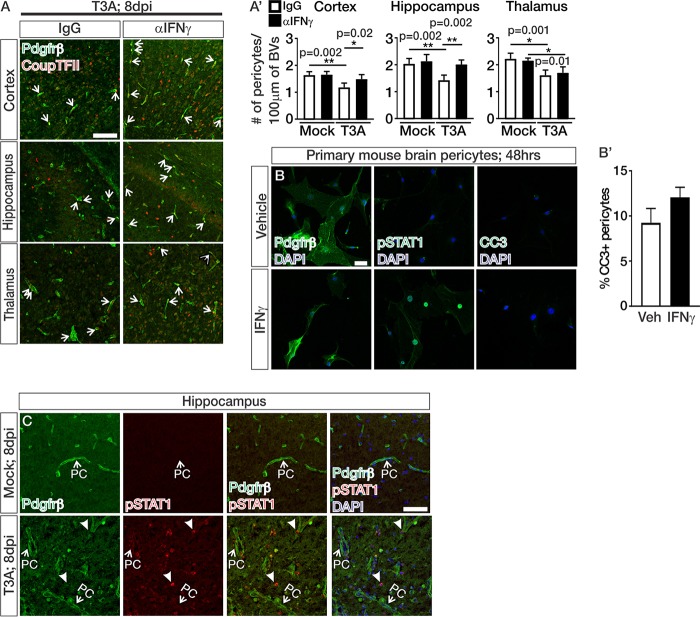
Neutralization of IFN-γ attenuates pericyte loss during CNS reovirus infection. (A and C) IHC images from the cortices of T3A-infected mice treated with IgG or αIFN-γ and immunostained for Pdgfrβ (green)- and CoupTFII (red)-expressing pericytes (A) or Pdgfrβ (green), pSTAT1 (red), and DAPI (blue) (C), where pericytes (PCs) are indicated by arrows. Closed arrows in panel C indicate pSTAT1^+^/Pdgfrβ^–^ cells. (A′) Quantification of Pdgfrβ^+^/CoupTFII^+^ pericytes/100 μm of blood vessels (BVs) in the cortices, hippocampi, and thalami of mock-infected (*n* = 4 animals/treatment) and T3A-infected (*n* = 5 animals/treatment) mice treated with IgG (white) or αIFN-γ (black). Analysis shows a significant improvement in the number of pericytes/100 μm of BV length in the cortices and hippocampi of T3A-infected mice treated with αIFN-γ. (B) Immunocytochemistry on primary brain pericytes after 48 h of vehicle of IFN-γ treatment immunostained for Pdgfrβ (green; left), pSTAT1 (green; middle) and CC3 (green; right) with DAPI (blue). (B′) Quantification of the percentage of CC3^+^ pericytes after 48 h of vehicle (white) or IFN-γ (black) treatment (*n* = 3 independent experiments). Scale bars, 50 μm.

We next tested whether reduced BBB leakage in T3A-infected mice treated with αIFN-γ correlated with improved pericyte number during CNS reovirus infection. Treatments with αIFN-γ attenuated pericyte loss in the cortex and hippocampus (Fig. 5A and A′). IFN-γ could directly cause the pericyte death we observed in T3A-infected animals; however, cultured brain pericytes exposed to IFN-γ for 48 h did not undergo apoptosis (Fig. 5B and B′). In addition, we did not detect evidence IFN signaling, as measured by pSTAT1 in Pdgfrβ-expressing pericytes at late stages of disease (Fig. 5C). Together, these data suggest that IFN-γ alone is not directly inducing cell death by activating IFN signaling in brain pericytes and that the improvement in pericyte density with αIFN-γ treatment is through an indirect, currently unknown mechanism.

### Neutralization of IFN-γ improves junctional organization in the brain vasculature during CNS reovirus infection.

**FIG 6:**
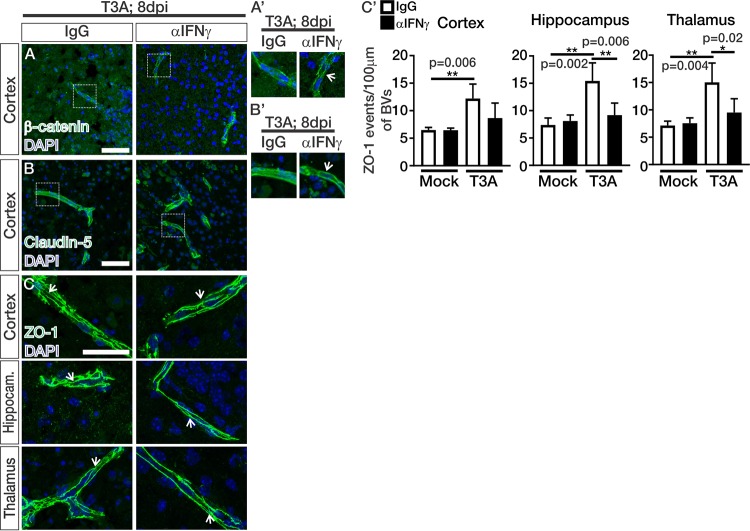
Neutralization of IFN-γ improves junctional organization in the brain vasculature during CNS reovirus infection. (A to C) IHC images from T3A-infected mice treated with IgG or αIFN-γ immunostained for the adherens junction adaptor protein β-catenin (green; cortex) (A), the tight junction protein claudin-5 (green; cortex) (B), and the adherens and tight junction adaptor protein ZO-1 (green; cortex, hippocampus, and thalamus) (C) with DAPI (blue). Junctions formed between vascular endothelial cells are indicated by arrows in ZO-1 staining (panel C). (A′ and B′) Enlarged images of β-catenin (A′) and claudin-5 (B′) indicate potential improvement in junctional organization in T3A-infected animals treated with αIFN-γ (arrows). (C′) Quantification of junctional organization of ZO-1 (as shown in Fig. 2F′) (see panel C) in the cortices, hippocampi, and thalami of mock-infected (*n* = 4 animals/treatment) and T3A-infected (*n* = 5 animals/treatment) mice treated with IgG (white) or αIFN-γ (black). Analysis shows significant improvement in ZO-1 crossing events/100 μm of blood vessel (BV) length in the hippocampi and thalami of αIFN-γ-treated mice. Scale bars, 50 μm.

We next assessed whether αIFN-γ treatments improved junctional organization in the brain vasculatures of animals infected with T3A. The protein expression levels of β-catenin (Fig. 6A), claudin-5 (Fig. 6B), and ZO-1 (Fig. 6C) within the vasculature did not appear to be overtly altered in T3A-infected mice treated with IgG or αIFN-γ. Enlarged images showed improved junctional zone localization for β-catenin and claudin-5 in animals treated with αIFN-γ (arrows; Fig. 6A′ and B′). Analysis of the ZO-1 junctional organization showed significant improvements in the hippocampi and thalami but not in the cortices in T3A-infected mice treated with αIFN-γ (Fig. 6C′). These studies show that αIFN-γ treatments improve junctional organization in the brain vasculature during T3A infection, and this improvement likely underlies the reduction in BBB leakage in T3A-infected animals treated with αIFN-γ (Fig. 4C).

### IFN-γ induces junctional disorganization through ROCK in brain endothelial cells.

**FIG 7:**
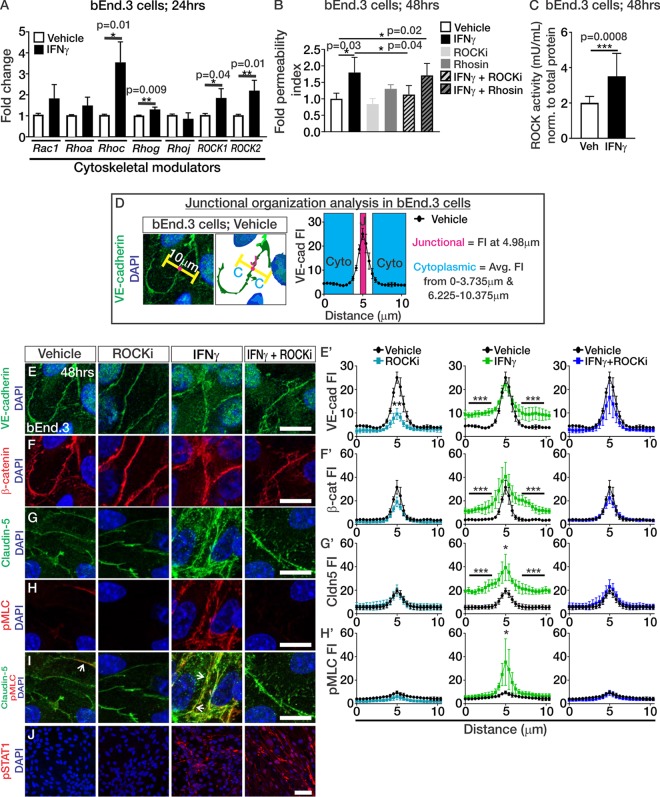
IFN-γ induces junctional disorganization through ROCK in brain endothelial cells. (A) Transcript expression of cytoskeletal modulators (*Rac1*, *Rhoa*, *Rhoc*, *Rhog*, *Rhoj*, *ROCK1*, and *ROCK2*) after 24 h of vehicle or IFN-γ treatment in bEnd.3 cultures (*n* = 3 independent experiments). (B) Fold changes in the permeability indices of bEnd.3 cultures (*n* = 4 independent experiments; after 5 min of NaF leakage) after 48 h of treatment with vehicle (white), IFN-γ (black), ROCK inhibitor Y-27632 (ROCKi; light gray), Rhosin (dark gray), IFN-γ+ROCKi (light gray and black stripes), and IFN-γ+Rhosin (dark gray and black stripes) show that IFN-γ requires ROCK activity to reduce barrier properties in brain endothelial cells. (C) ROCK activity assay based off phosphorylation of myosin phosphatase (normalized to total protein concentration μg/μl) in bEnd.3 cells after 48 h of vehicle (white; *n* = 6) and IFN-γ (black; *n* = 6) treatment. (D) Schematic to depict junctional organization analysis by measuring fluorescent intensity of junctional proteins over ∼10-μm measurements across junctions (pink area) and in the cytoplasm (blue area) of two neighboring cells where junctions were consistently aligned at 4.98 μm. (E to J) Immunocytochemistry of VE-cadherin (green) (E), β-catenin (red) (F), claudin-5 (green) (G), phospho-myosin light chain (pMLC; red) (H), claudin-5 (green) and pMLC (red) (I), pSTAT1 (red) with DAPI (blue) (J) after 48 h of vehicle or IFN-γ exposure with or without ROCKi in bEnd.3 cells. (E′ to H′) Fluorescence intensity (FI) plots to depict localization (based off analysis depicted in panel D) of VE-cadherin (E′), β-catenin (F′), claudin-5 (G′), and pMLC (H′) after 48 h of vehicle (black), ROCKi (teal), IFN-γ (green), or IFN-γ+ROCKi (blue) exposure in confluent bEnd.3 cells. Statistical analysis for significant findings in protein localization in these plots was calculated from data graphed and compared via ANOVA with Tukey′s *post hoc* analysis, where the junctional fluorescence intensity is considered the fluorescence intensity at 4.98 μm and the cytoplasmic fluorescent intensity is the average fluorescent intensity from 0 to 3.735 μm and from 6.225 to 10.375 μm, as depicted in panel D (*, *P* < 0.05; **, *P* < 0.01; ***, *P* < 0.001). Three independent experiments were performed for each treatment condition. Scale bars: E to I, 25 μm; J, 50 μm.

We next assessed whether IFN-γ is directly causing endothelial junctional disorganization and how this may reduce barrier integrity in brain endothelial cells. The junctional disorganization we observed points to cytoskeletal events occurring in the brain vasculature during T3A infection. Consistent with this idea, IFN-γ activation of Rho kinase (ROCK) is hypothesized to initiate cytoskeletal rearrangements that disrupt TJ connections in epithelial cells (28). We first tested this idea in brain endothelial cells by assessing whether IFN-γ directly alters gene expression of cytoskeletal modulators known to regulate vascular junctional integrity such as Rac1-GTPases and Rho-GTPases, as well as ROCKs (15, 29–31). We found that IFN-γ treatment of cultured bEnd.3 cells significantly induced the expression of *Rhoc*, *Rhog*, *Rock1*, and *Rock2* (Fig. 7A). Treatment with IFN-γ elevated the expression of the known IFN target genes *Stat1* (*P* = 0.07; vehicle means ± the standard errors of the mean [SEM]: 1.04-fold change ± 0.01155 [*n* = 3]; IFN-γ means ± the SEM: 26.97-fold change ± 10.77 [*n* = 3]) and *Ifitm3* (*P* = 0.02; vehicle means ± the SEM: 1.007-fold change ± 0.003 [*n* = 3]; IFN-γ means ± the SEM: 4.583-fold change ± 0.88 [*n* = 3]), verifying activation of IFN signaling in these experiments. Since we observed upregulation of Rho-GTPases and ROCKs, we next tested whether IFN-γ requires their activity to reduce barrier function in brain endothelial cells. To test this, we performed permeability assays on bEnd.3 cells exposed to IFN-γ with or without the Rho and ROCK activity inhibitors Rhosin and Y-27632 (ROCKi), respectively. Rhosin is effective for inhibiting RhoA and RhoC-GTPases without affecting the activity of other GTPases such as Rac1 (32). Y-27632 is a selective inhibitor for both ROCK1 and ROCK2 by competing with their ATP binding domains (33, 34). Interestingly, bEnd.3 cultures treated with IFN-γ displayed increased permeability; however, inhibition of ROCK activity (ROCKi), but not Rho-GTPase activity (Rhosin), attenuated the IFN-γ-mediated leakage of NaF (Fig. 7B). In addition, IFN-γ significantly induced ROCK kinase activity in the bEnd.3 cells (Fig. 7C) indicating that IFN-γ directly regulates ROCK activity and requires it to induce permeability in brain endothelial cells.

We next assessed the impact of IFN-γ and ROCK activity on junctional organization in brain endothelial cells. After 48 h of vehicle or IFN-γ with or without ROCKi (Y-27632) exposure in bEnd.3 cells, we measured the fluorescence intensity profile of the junctional proteins along ∼10-μm distances that spanned the junctions and the cytoplasm of two neighboring cells, where junctions were consistently aligned at 4.98 μm along the measurements (the method and calculations for analysis are depicted in Fig. 7D). We utilized Y-27632 to inhibit ROCK1/2 activity in these experiments since it has been used to attenuate BBB permeability in mice with genetic causes of vascular malformations (30). IFN-γ treatment increased the cytoplasmic fluorescent intensity of VE-cadherin (Fig. 7E and E′), β-catenin (Fig. 7F and F′), and claudin-5 (Fig. 7G and G′). Junctional localization (the fluorescence intensity at 4.98 μm) was not reduced but rather increased in claudin-5 junctional fluorescence following IFN-γ treatment (Fig. 7G and G′). Interestingly, junctional localization of the ROCK substrate, pMLC (35), was significantly increased after IFN-γ treatment (Fig. 7H and H′) and colocalized with claudin-5 (Fig. 7I), a phenomenon that has been reported to weaken cell-cell interactions (36). Inhibition of ROCK activity during IFN-γ exposure blocked the cytoplasmic mislocalization of junctional proteins (Fig. 7E, G, and E′ to G′) and the junctional localization of pMLC (Fig. 7H and H′). Of note, VE-cadherin junctional localization was reduced following ROCKi inhibition (Fig. 7E and E′). However, junctional localization of β-catenin (Fig. 7F and F′) or claudin-5 (Fig. 7G and G′) was not altered, suggesting that inhibition of ROCK activity alone does not overtly affect junctional protein localization. These data demonstrate that IFN-γ disrupts normal junctional protein localization through ROCK activity.

### IFN-γ induces ROCK-mediated cytoskeletal contractions and cell-cell separations in brain endothelial cells during CNS reovirus infection.

**FIG 8:**
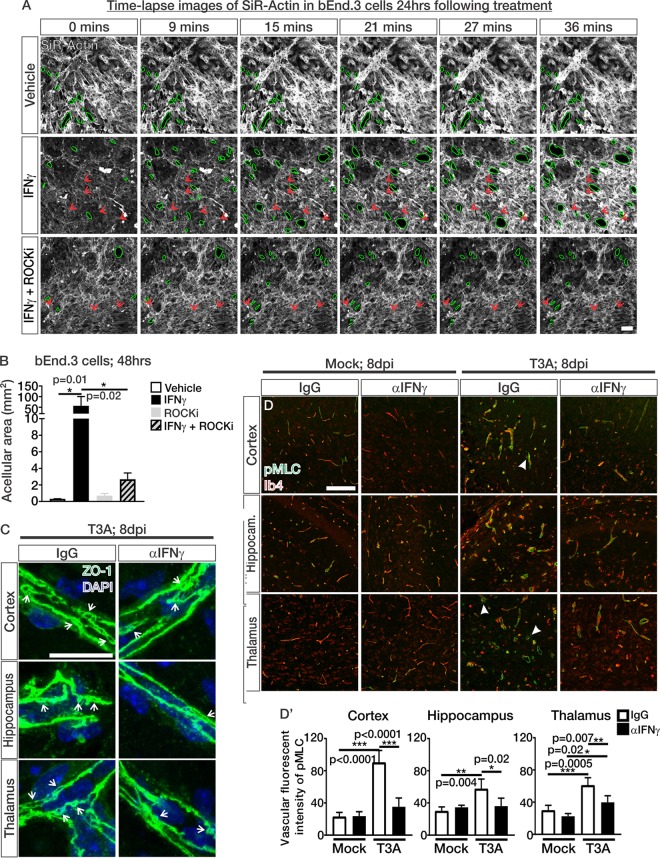
IFN-γ induces ROCK-mediated cytoskeletal contractions and cell-cell separations in brain endothelial cells during CNS reovirus infection. (A) Time-lapse confocal imaging over 36 min of SiR-Actin-labeled actin (white) in bEnd.3 cells after 24 h of vehicle, IFN-γ, or IFN-γ+ROCK inhibitor (ROCKi; Y-27632) treatment. Green outlines indicate regions of acellular formations/loss of cell-cell contact. Red arrows point to actin stress fibers in IFN-γ and IFN-γ+ROCKi images. Scale bar, 50 μm. (B) Quantification of acellular area (mm^2^) based off phalloidin staining normalized to the total number of cells per field shows a significant increase in the acellular area in bEnd.3 cultures after 48 h of IFN-γ treatment; however, treatment with the ROCKi blocked this effect (*n* = 3 independent experiments). (C) Enlarged images of ZO-1-stained BVs show potential junctional separations (arrows) in T3A-infected mice; however, they appear to be reduced in mice treated with αIFN-γ. Scale bar, 25 μm. (D) IHC images of the cortices, hippocampi, and thalami of mock- and T3A-infected mice treated with IgG or αIFN-γ immunostained for phospho-myosin light chain (pMLC; green) and Ib4 (red). Closed arrows indicate pMLC expression in nonvascular cells, presumably migratory immune cells like microglia and macrophages. (D′) Quantification of the fluorescence intensity of pMLC in the Ib4^+^ vasculature of mock-infected (*n* = 4 animals/treatment) and T3A-infected mice shows that there is a reduction in pMLC presence in the vasculature of T3A-infected mice treated with αIFN-γ (*n* = 5 animals/treatment) compared to T3A-infected mice treated with IgG (*n* = 4 animals/treatment). Scale bar, 50 μm.

To understand how IFN-γ and ROCK activity work together to alter junctional organization in brain endothelial cells, we assessed actin cytoskeletal dynamics using time-lapse confocal imaging of SiR-Actin in confluent bEnd.3 cultures. These studies revealed major and rapid separations (<40 min) occurring between cells after 24 h of IFN-γ exposure compared to vehicle-treated cultures (acellular areas outlined in green; Fig. 8A and see Video S1 in the supplemental material). Actin stress fibers, which are aggregates of actin fibers that are utilized for cytoskeletal contractions (31, 35), were apparent in all samples treated with IFN-γ, even in the presence of ROCKi; however, they rapidly contracted in IFN-γ samples (red arrows; Fig. 8A and Videos S2 and S3), as evidenced by their increase in fluorescence intensity over time (red arrows; Fig. 8A). Major cell-cell separations were not as apparent in IFN-γ+ROCKi-treated cultures and fluorescent intensity of the actin stress fibers remained stable throughout time-lapse imaging experiments indicating lack of actin stress fiber contractions in these treatment conditions (red arrows: Fig. 8A and see Video S3). The acellular area, based on phalloidin staining, after 48 h of treatment was significantly increased in bEnd.3 cells treated with IFN-γ however this was blocked when ROCK activity was attenuated (Fig. 8B). These results, taken together, demonstrate that IFN-γ requires ROCK activity to reduce barrier integrity in bEnd.3 cells through actin cytoskeletal contractions, and this coincides with cell-cell separations. This mechanism likely underlies the junctional protein mislocalization and barrier leakage in bEnd.3 cells exposed to IFN-γ (Fig. 7).

Upon closer evaluation of ZO-1 staining in T3A-infected animals, we noted a zipper-like appearance of ZO-1-labeled junctions suggestive of loss of contact between adjacent endothelial membranes (arrows; Fig. 8C). These junctional separation events appeared less frequently in T3A-infected mice treated with αIFN-γ (arrows; Fig. 8C). These events are potentially analogous to cell-cell separation events caused by increased ROCK activity upon IFN-γ treatment in culture brain endothelial cells. Therefore, we next looked for evidence of ROCK activation within the brain vasculature. Indeed, we observed significant increases in vascular pMLC expression in T3A-infected mice treated with IgG, and this was attenuated in T3A- infected mice treated with αIFN-γ (Fig. 8D and D′). In addition to the vasculature, pMLC expression was occasionally noted in other cell types, which based on morphology appear to be migratory immune cells such as microglia and macrophages (closed arrows; Fig. 8D). Taken into consideration with our culture experiments, these results suggest that IFN-γ induces ROCK activation in the brain vasculature, resulting in cytoskeletal contractions that affect junctional integrity and induce cell-cell separations. This mechanism likely contributes to impaired BBB integrity and results in BBB leakage during CNS reovirus infection.

## DISCUSSION

**FIG 9:**
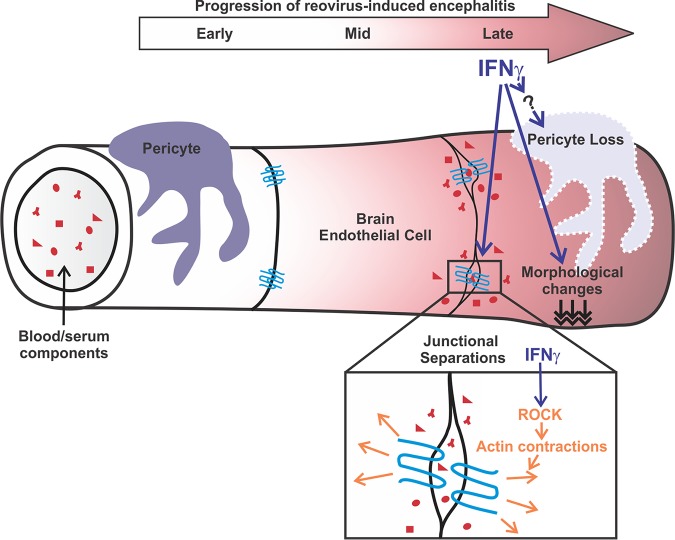
IFN-γ alters junctional integrity via ROCK, resulting in BBB leakage in experimental viral encephalitis. Our comprehensive analysis reveals that BBB leakage occurs at late stages of CNS reovirus infection. We found that BBB leakage correlates with morphological changes, reductions in pericyte numbers, and disorganization of junctions in the brain vasculature. Identification of endothelial-IFN signaling through transcriptomic analysis in combination with *in vitro* and *in vivo* investigations revealed IFN-γ as an inducer of BBB leakage during CNS reovirus infection, most notably through ROCK-mediated cytoskeletal contractions in brain endothelial cells that disrupt junctional organization and cell-cell connections. Furthermore, we found that neutralization of IFN-γ during infection also attenuates pericyte loss. However, our studies do support a direct role for IFN signaling in inducing pericyte death during infection and therefore may be working with other factors to affect pericyte populations.

Our comprehensive analysis of BBB properties has identified cellular and molecular events that occur in the brain endothelium contributing to BBB leakage during CNS reovirus infection. We found that IFN signaling, mediated by IFN-γ, disrupts barrier integrity by inducing cell-cell separations through ROCK-mediated cytoskeletal contractions and mislocalization of junctional proteins in brain endothelial cells. Furthermore, our work shows that IFN-γ contributes to the loss of pericytes, potentially through an indirect mechanism, during CNS reovirus infection. These alterations in BBB properties mediated by IFN-γ are major contributors to BBB leakage during CNS reovirus infection (Fig. 9).

BBB leakage during virus-induced encephalitis is well documented (3); however, the underlying cellular and molecular mechanisms that induce BBB leakage are not well understood. We found that BBB leakage occurs at late stages of reovirus-induced encephalitis, a time point that corresponds with substantial cell death and when significant innate immune responses are known to be occurring (37). Concurrent with leakage, we document morphological changes in the vasculature and substantial alterations in junctional organization—events that are consistent with the loss of pericytes (8, 23). It is possible that these morphological changes in the vasculature alter junctional organization and destabilize intracellular connections. Alternatively, loss of junctional connections may compromise the uniform blood vessel diameter, though which event drives which is difficult to discern. Regardless, it is likely that these cellular alterations together are the main underlying cause of the BBB leakage that occurs in this mouse model of virus-induced encephalitis. Further, it is important to note that disorganization of junctions in the vasculature has been observed in a number of virus-induced encephalitis mouse models, including West Nile and alphavirus encephalitis models, as well as lipopolysaccharide-mediated inflammatory instances (15–17). Therefore, junctional disorganization may be a common mechanism underlying BBB leakage across encephalitis models.

Disruptions to endothelial cytoskeletal organization have been speculated to be an underlying mechanism for altering junctional integrity and BBB leakage either by relaxing or contracting the intracellular interactions (4, 29–31, 38, 39). Proteomic studies of brain endothelial cells following La Crosse viral infection found dysregulation in many proteins involved in cytoskeletal rearrangements/organization (40). A number of studies in other CNS diseases have pointed to cytoskeletal contraction via activation of ROCK as a driving factor behind BBB leakage and disrupting junctional integrity (29, 30, 39). Our data have identified ROCK as specifically mediating cytoskeletal contractions in cultured brain endothelial cells downstream of IFN-γ. Through transcriptomic analysis of brain endothelial cells, we identified IFN signaling as a major pathway that is activated in the brain vasculature following CNS reovirus infection. IFN signaling, well established for its role in regulating antiviral responses, is not well known to regulate cytoskeletal processes. However, IFN-γ, specifically, is known to be barrier disruptive during viral infection (11, 13). Experiments performed by Chai et al. (11) found that blockage of IFN-γ (using αIFN-γ) in cultured mouse brain microvascular endothelial cells treated with brain extracts from rabies virus-infected animals improved TJ protein expression and barrier properties. This study supports the idea that IFN-γ is barrier disruptive in brain endothelial cells exposed to disease tissue lysate. Our work builds significantly on these studies, identifying ROCK and cytoskeletal contractions as key mediators of IFN-γ disrupting barrier integrity in brain endothelial cells.

Our *in vitro* studies strongly point to IFN-γ acting through ROCK to induce cytoskeletal contractions. We believe this alters junctional protein localization resulting in loss of cell-cell connections. In cultured monolayers this appears as pores/acellular regions and, *in vivo*, we suspect this manifests as periodic separations in ZO-1-labeled junctions. These separations do not result in complete loss in endothelial cell-cell contact *in vivo*, as indicated by the absence of hemorrhage at these stages of disease. Instead, we believe this erodes the extent of TJ contact between cells that is required to limit paracellular movement of molecules across the brain endothelium. ROCK is known to induce cytoskeletal contractions either through direct phosphorylation of MLC or phosphorylation and subsequent deactivation of MLC phosphatase, a negative regulator of pMLC contractile activity (35). It is unclear whether ROCK is acting directly by phosphorylating MLC or indirectly by inactivating MLC phosphatase; however, pMLC was present after IFN-γ treatment and reduced when ROCK activity was inhibited. Here, we demonstrate evidence of ROCK activation (pMLC) in the brain vasculature, along with the junctional separations during reovirus infection. This suggests that cytoskeletal contractions mediated by ROCK activation are occurring in the brain vasculatures of infected mice downstream of IFN-γ signaling within the brain endothelium. The IFN-γ-ROCK mechanism of BBB breakdown we describe for reovirus-induced encephalitis is potentially contributing to the brain endothelial junction disorganization observed in other viral encephalitis and inflammatory instances (15–17). Inhibition of ROCK activity using Y-27632 has been successfully used in genetic models of cerebral cavernous malformations to block elevated endothelial ROCK activity and improve vascular integrity (30). However, Y-27632’s potent anti-inflammatory effects (41) are likely to be problematic for use in mouse models of viral encephalitis to attenuate BBB leakage since they could impair the inflammatory response that is needed to counter viral infection. It is unknown at this time whether IFN-γ regulates ROCK through canonical IFN-γ/IFNGR1/STAT1 signaling or through noncanonical/indirect mechanisms. IFN-γ derived from T cells is the likely source that is inducing IFN signaling, ROCK activity, and BBB leakage. CD3^+^ and CD8^+^ T cells were present in the neural parenchyma and along the vascular wall during late stages of reovirus infection. As noted, there were some instances of perivascular cuffing with high concentrations of T cells (and potentially IFN-γ) surrounding the vasculature. However, alterations to the BBB were readily apparent in the absence of perivascular cuffing. Regardless, our data would suggest that T cells in the neural parenchyma or along the vascular wall are a source of IFN-γ, inducing BBB leakage through ROCK activity and junctional disorganization.

In these studies, we observed that anti-IFN-γ-induced improvements in the BBB correlate with reductions in cell death. Possibly, blocking IFN-γ attenuates immune cell release of cell death factors in the CNS during reovirus infection. In addition, IFN-γ has been shown to be neurotoxic in a viral *déjà vu* mouse model induced by lymphocytic choriomeningitis virus (42) and in cultured mouse cortical neurons (43); thus, blockage of IFN-γ during T3A infection may have attenuated these effects on neurons.

We also found that pericyte numbers are reduced at late stages of reovirus infection, a phenomenon that, to our knowledge, has not been looked at so far in viral encephalitis mouse models. Pericytes are known to be sensitive to inflammation, and reductions in pericyte coverage occur in many CNS diseases associated with BBB dysfunction (44–50). Thus, is it likely that the death and clearance of pericytes that we observed is part of encephalitis disease pathogenesis. Although blockage of IFN-γ improved pericyte coverage in reovirus-infected mice, 48 h of IFN-γ exposure does not induce pericyte death in culture, and we did not find evidence of active IFN-STAT signaling in pericytes *in vivo*. This suggests that *acute* pericyte IFN signaling does not contribute to pericyte death. It is possible that retention of the pericyte population in reovirus-infected animals treated with αIFN-γ is due to (i) improvement in the overall vasculature, since pericytes require endothelium-derived trophic factors such as PDGF-B (51) or, as discussed previously, (ii) attenuation of IFN-γ-mediated release of cell death or cytotoxic factors such as tumor necrosis factor alpha and interleukin-1β that have been shown to induce pericyte death (52, 53).

Loss of BBB integrity has been described in a number of viral encephalitis animal models (HIV, Zika virus, Japanese encephalitis virus, La Crosse virus, and West Nile Virus) (3, 40, 54) and human cases of HIV-induced encephalitis (55, 56). Here, we identified endothelial-IFN-γ signaling as a contributor to BBB leakage. IFN-γ is a major cytokine upregulated in most CNS viral infections. Therefore, our findings with regard to IFN-γ-induced junctional disorganization via ROCK and pericyte loss are potentially broadly applicable to encephalitis caused by other viruses. There are few treatment options for virus-induced encephalitis in patients; therefore, targeting the BBB breakdown that occurs in encephalitis is an attractive option. Our studies revealing the timing of breakdown (e.g., late in disease) and the cellular (e.g., junctional disorganization and pericyte loss) and molecular changes (e.g., IFN-γ-induced activation of cytoskeletal modulator ROCK in the brain vasculature) provide a much more complete picture of BBB breakdown in viral encephalitis. This could aid in the development of therapeutic strategies to ameliorate barrier disruption in this disease and improve patient survival.

## MATERIALS AND METHODS

### Animals and reovirus infections.

Mice used for experiments here were housed in specific-pathogen-free facilities approved by the AALAC and were handled in accordance with protocols approved by the University of Colorado Anschutz Medical Campus IACUC committee. Swiss Webster (Jackson Laboratory), *Ai14-flox* (Jackson Laboratory), and *Cdh5cre^ERT2^* (obtained from Ralf Adams; bred to a Swiss Webster background) mice were used in these studies. All pups were intracranially infected with 1,000 PFU of reovirus serotype 3 strain Abney (T3A) in 10 μl of PBS or 10 μl of PBS (mock) at P2. To activate Cre-mediated recombinase activity in *Cdh5cre^ERT2/+^*; *Ai14^fl/+^* pups prior to mock or T3A infection, pups were injected intraperitoneal with 50 μl of tamoxifen (Sigma) dissolved in corn oil (Sigma; 1 mg/ml) at P0 and P1 to express Tdtomato within the vasculature. To measure BBB leakage, mice were injected intraperitoneal with 25 μl of 0.25% Biocytin-TMR (Thermo Fisher) for 2 h prior to isolation of tissue for immunohistochemistry at their respective time point. For IFN-γ neutralization experiments, Swiss Webster or *Cdh5cre^ERT2/+^*; *Ai14^fl/+^* pups were given intraperitoneal injections of a monoclonal antibody to block IFN-γ, rat anti-mouse IFN-γ (clone XMG1.2), or rat IgG (10 μg/day; BioLegend) from 4 to 7 dpi. Mice were then weighed, and brains were isolated at 8 dpi for immunohistochemical or transcriptional analyses, or mice were allowed to proceed to 9 or 10 dpi and either succumb or euthanized due to disease (survival experiments).

### Immunohistochemistry.

For experiments investigating BBB leakage, the brain vasculature, and BBB properties throughout reovirus infection, mock- and T3A-infected mice (3, 5, or 8 dpi; *n* = 4 animals/time point/mock or T3A cohort) were anesthetized, and transcardiac perfusions were performed with PBS, followed by 4% paraformaldehyde. The skull was removed, and the brain was dissected, cryoprotected with 20% sucrose in PBS, and subsequently frozen in OCT. Similarly, brains were isolated and fixed in experiments where IFN-γ was blocked in mock- and T3A-infected mice (mock, IgG or αIFN-γ [*n* = 4 animals]; T3A, IgG or αIFN-γ [*n* = 5 animals]). Tissue was cryosectioned in 12-μm increments. Immunohistochemistry was performed on tissue sections as described previously (57, 58) using the following antibodies: mouse anti-β-catenin (1:100; Cell Signaling), rabbit anti-CD3 (1:100; Abcam), rat anti-CD8α (1:50; Novus), rat anti-CD13 (1:100; Bio-Rad), anti-claudin-5^488^ (1:100; Invitrogen), rabbit anti-cleaved caspase 3 (1:100; Cell Signaling), mouse anti-collagen IV (1:250; Sigma), mouse anti-CoupTFII (1:100; R&D Systems), rabbit anti-Iba1 (1:100; Wako), rabbit anti-Pdgfrβ (1:100; Cell Signaling), mouse anti-phospho-myosin light chain-2 (1:100; Cell Signaling), rabbit anti-pSTAT1 (1:100; Cell Signaling), mouse anti-σ3 (1:200), and mouse anti-ZO-1 (1:100; Thermo Fisher). After incubation with primary antibody(s), sections were incubated with appropriate Alexa Fluor-conjugated secondary antibodies (Invitrogen), Alexa Fluor 633-conjugated isolectin-B4 (Invitrogen), and DAPI (4′,6′-diamidino-2-phenylindole; Invitrogen). Biocytin-TMR fluorescence was detected due to its endogenous fluorescence (∼554/581 nm) or staining with streptavidin-594 (1:500; Thermo Fisher) in samples that received antigen retrieval. Immunofluorescence images were captured using a Nikon i80 research microscope with a Cool-Snap CCD-cooled camera or a Zeiss 780 LSM confocal microscope.

### Quantitative analysis of immunohistochemistry.

Morphological analysis of blood vessels was determined by measuring the diameters of the collagen-IV-labeled blood vessels (μm; three to five ×200 images/region/animal; Nikon image analysis software). For BBB leakage analysis, ImageJ threshold analysis was performed on ×100 images (three to five images/region/animal) of Biocytin-TMR puncta and normalized to total number of DAPI^+^ cells per image using threshold analysis. To quantify the pericyte coverage, the number of Pdgfrβ/CoupTFII^+^ cells in each image was quantified and divided by the total length of Ib4^+^ or Tdtomato^+^ blood vessels (μm) and then multiplied by 100 to achieve the number of pericytes per 100 μm of blood vessel using Zen software (three to five ×200 confocal images/region/animal). To determine junctional organization images were analyzed for the number of ZO-1 crossings through the center of longitudinal blood vessels (analysis as depicted in Fig. 2F′ was performed using Zen software on three to five ×400 confocal images/region/animal). The number of ZO-1 intersection events was normalized to the total blood vessel length (μm; identified by Ib4 staining or Tdtomato expression) per image and multiplied by 100 to achieve the number of ZO-1 crossing events per 100 μm of blood vessel. The number of endothelial cells with active IFN signaling was determined by counting the number of pStat1-endothelial nuclei divided by the total number of Ib4^+^/Tdtomato^+^ endothelial cells per image using Zen software (three 20× images/region/animal). ImageJ threshold analysis was also performed on cleaved caspase 3 (CC3) (three 10× images/region/animal) and normalized to total number of DAPI^+^ cells per image using threshold analysis to get CC3 density. The fluorescence intensity of pMLC in the brain vasculature was determined using Zen contour functions to measure the fluorescent intensity specifically within the Ib4^+^ vasculature (three 20× images/region/animal).

### Fluorescence-activated cell sorting of brain endothelial cells.

To isolate brain endothelial cells from mock (*n* = 2)- and T3A (*n* = 2)-infected mice, brains were isolated from animals at 6 dpi, and meninges were dissected from the brains. Forebrain and midbrain regions were minced with forceps and digested in Hanks balanced salt solution (HBSS) with calcium and magnesium supplemented with 2% bovine serum albumin (BSA), 300 mg/ml glucose, 10 mg/ml collagenase type 2 (Worthington), and 10 mg/ml DNase for 15 to 20 min at 37°C. Samples were centrifuged at 1,000 rpm for 5 min, and the pellet was resuspended in HBSS with calcium and magnesium supplemented with 1% BSA. Samples were then filtered and stained with DAPI to eliminate dead cells during cell sorting. Samples were then run on Moflo Astrios EQ by the University of Colorado Center Flow Cytometry Core. Tdtomato^+^ cells were collected for downstream transcriptional analysis via RNA-seq. Tdtomato^–^ cells were collected and assessed for the expression of IFN ligands (the methods are described below).

### RNA-seq and transcriptome analysis on brain endothelial cells.

After fluorescence-activated cell sorting of the brain endothelial cells, the samples were lysed, and RNA was isolated using an RNeasy microkit (Qiagen). RNA quality was analyzed using a Agilent 2100 Bioanalyzer and a 2200 Tape Station. Samples used for RNA-seq had RINe (RNA integrity number equivalent) values between 8.1 and 9.1. A total of 10 to 100 ng of total RNA was used to prepare Illumina HiSeq libraries according to the manufacturer’s instructions for the TruSeq stranded RNA kit (Illumina, Inc.). The mRNA template libraries were then sequenced as single-pass 50-bp reads on the Illumina HiSeq4000 platform at the University of Colorado’s Genomics and Sequencing Core Facility. Derived sequences were analyzed by applying a custom computational pipeline consisting of the open-source gSNAP, Cufflinks, and R for sequence alignment and ascertainment of differential gene expression (59). In short, reads generated were mapped to the mouse genome by gSNAP (60), expression (FPKM) was derived by Cufflinks (61), and differential expression was analyzed using analysis of variance (ANOVA) in R. Genes significant at a false discovery rate of <0.05 were subjected to Ingenuity Pathway Analysis (Qiagen) to identify pathways of interest that were activated in brain endothelial cells following T3A infection. With an *n* value of 2 for mock- and T3A-infected mice in these studies, we have a power of 93% to detect a 2-fold change with a *P* value of 0.05.

### Transcriptional analysis on tissue.

After treatments with IgG or αIFN-γ in mock- and T3A-infected mice, brains were hemisected, and the cortices, hippocampi, and thalami were dissected. RNA was isolated from each region to assess the virus titers via PCR was quantified as previously described (62), utilizing viral standards of the genomic region plasmid amplified. Primers RV3F (5′-GCTATGTCATATTTCCATCCG-3′) and RV4R (5′-CATATGACTACCACTTTCCCG-3′) were used to quantify virus targeted the L1 region of the reovirus genome. In the cortices, hippocampi, and thalami of mock- and T3A-infected mice treated with IgG, *Ifna*, *Ifnb*, and *Ifng* transcript expression was assessed using the following primers: *Ifna* forward, GACTCATCTGCTGCATGGAA; *Ifna* reverse, CAGGGGCTGTGTTTCTTCTC; *Ifnb* forward, TTACACTGCCTTTGCCATCC; *Ifnb* reverse, TTGAGGACATCTCCCACGTC; *Ifng* forward, CGGCCTAGCTCTGAGACAAT; and *Ifng* reverse, GTCACCATCCTTTTGCCAGT.

### bEnd.3 cell cultures and treatments.

The mouse brain endothelioma cell line (bEnd.3) were obtained from the American Type Culture Collection (CRL-2299). All experiments were performed on cells from passages 5 to 15, and cells were grown in Dulbecco minimal essential medium supplemented with endothelial cell growth factor (Sigma), 4.5 g/liter glucose, 1.5 g/liter sodium bicarbonate, 4 mM l-glutamine (Invitrogen), 10% fetal bovine serum (FBS; Invitrogen), and penicillin (0.0637 g/liter)-streptomycin (0.1 g/liter). Cells were treated with either dimethyl sulfoxide (DMSO; vehicle), 100 ng/ml IFN-γ (R&D), or 2 μg/ml poly(I:C) (R&D). To inhibit Rho and ROCK activity, bEnd.3 cells were treated with 150 nM Rhosin (300 nM Rhosin appeared to be toxic to the bEnd.3 cells; Tocris) and 300 nM Y-27632 (ROCKi; Tocris).

### Permeability assays.

bEnd.3 cells were plated onto collagen-coated 0.4-μm transwell plates (Corning). Cells were allowed to reach full confluence and maintained there for at least 3 days to mature junctions. The cells were serum starved overnight and then treated with vehicle, IFN-γ, poly(I:C), Y-27632, or Rhosin for 48 h. Wells were then washed with Krebs Ringer solution (Alfa Aesar) three times. Abluminal wells (lower well) were loaded with Krebs Ringer solution, and a luminal well (upper well) was loaded with 100 μg/ml sodium fluorescein (NaF). The, 5 μl of the luminal well was isolated and diluted in 200 μl of Krebs Ringer solution as a starting fluorescence. In experiments involving IFN-γ and poly(I:C), leakage of NaF was assessed after 60 min where 5 μl of the abluminal well was isolated and also diluted to measure the leakage of NaF. In experiments comparing IFN-γ with and without Y-27632 and Rhosin, leakage of NaF was assessed after 5, 10, 30, and 60 min to accurately identify differences in NaF leakage between the different treatment conditions. Leakage of NaF is shown after 5 min in Fig. 5E; however, the trend was similar at all time points. The fluorescence intensity was measured by using a BioTek plate reader with excitation at 485 and emission reading at 530. The equation P_NaF_ = (RFU_Lower_/RFU_Upper_)(V)(1/time)(1/area) was used to calculate the permeability coefficient. All experiments (a minimum of *n* = 3 independent experiments) were performed in triplicate, and permeability fold changes were calculated between control and treatment conditions for each experiment. After the permeability assays, the wells were washed three times with Krebs Ringer solution, and the wells were stained for some downstream immunocytochemical analysis.

### Transcriptional analysis in bEnd.3 cultures.

Transcriptional analysis on BBB gene expression in bEnd.3 cells was performed after 24 h of treatment with vehicle or IFN-γ (*n* = 3 independent experiments with technical replicates in triplicate for each treatment condition). RNA was isolated by using an RNeasy microkit (Qiagen). cDNA was synthesized using iScript reverse transcription supermix (Bio-Rad), and the expression of BBB genes, cytoskeletal modulators, and IFN target genes were analyzed using SYBR green and a Bio-Rad CFX96 real-time PCR detection system. ΔΔ*C_T_* values were calculated. Expression levels for transcripts of interest were normalized to *Cyclophilin-A* (*CyA*) expression. The following primer sequences were used: *CyA* forward, GAGCTGTTTGCAGACAAAGTTC; *CyA* reverse, CCCTGGCACATGAATCCTGG; *Ifitm3* forward, CCCCCAAACTACGAAAGAATCA; *Ifitm3* reverse, ACCATCTTCCGATCCCTAGAC; *Rac1* forward, AGTGTGTGGTGGTGGGAGAC; *Rac1* reverse, GTAGGAGAGGGGACGCAATC; *Rhoa* forward, GTGGATGGGAAGCAGGTAGA; *Rhoa* reverse, GGGCACATTTGGACAGAAAT; *Rhoc* forward, AGCCCTGACAGTTTGGAAAA, *Rhoc* reverse: CCAAAGGCACTGATCCTGTT; *Rhog* forward: GCATCAAGTGTGTGGTGGTG; *Rhog* reverse, AGGCGGTCATATTCCTCCTG; *Rhoj* forward, GAAGCCGAGTGACAATCCAT; *Rhoj* reverse, GCGTTAAGGTGCTTGGGTAA; *Rock1* forward, CCTGATAACATGCTGCTGGA; *Rock1* reverse, AATACCCCAACTGACCACCA; *Rock2* forward, TTAACCCCACAGCAGAGTGC; and *Rock2* reverse, GCCAACAGCCACTATTTCCA. Transcriptional analysis on IFN target gene expression in bEnd.3 cells was performed after 24 h of treatment with vehicle or poly(I:C) (*n* = 3). The following primers were used for these experiments: *Actb* forward, CTAGGCACCAGGGTGTGAT; *Actb* reverse, TGCCAGATCTTCTCCATGTC; *STAT1* forward, TCACAGTGGTTCGAGCTTCAG; *STAT1* reverse, GCAAACGAGACATCATAGGCA; *Ifitm3* forward, CCCCCAAACTACGAAAGAATCA; and *Ifitm3* reverse, ACCATCTTCCGATCCCTAGAC.

### ROCK activity ELISA.

bEnd.3 cells were plated onto 6-well plates and allowed to reach 80 to 90% confluence. Cells were serum starved overnight and then treated with vehicle or IFN-γ. After 48 h, the cells were lysed in radioimmunoprecipitation assay buffer, and the ROCK activity was measured by assessing the phosphorylation of myosin phosphatase via activity enzyme-linked immunosorbent assay (EMD Millipore) according to protocol specifications. Appropriate positive (recombinant ROCK II) and negative controls (ROCK inhibitor Y-27632) were included. ROCK activity (mU/μl) in each sample was normalized to the total protein concentration (μg/μl), quantified using a Pierce BCA assay.

### Immunocytochemistry and analysis of bEnd.3 cultures.

After vehicle and IFN-γ with or without Y-27632 (ROCKi) treatments and permeability assays, cells plated on the inner well of the transwell plates were fixed with 4% paraformaldehyde (PFA) for phalloidin staining or 100% methanol for pSTAT-1 staining for 10 min. The wells were then incubated with rabbit anti-pSTAT1 (1:100; Cell Signaling) or stained with phalloidin-568 (1:100; Thermo Fisher) for 1 h at room temperature. The cells were then incubated the appropriate Alexa Fluor secondary antibody and DAPI. After several washing steps, the inner well membrane was excised and mounted for imaging. Immunofluorescent images were captured using a Zeiss 780 LSM confocal microscope. Zen imaging software was used to assess the acellular area of bEnd.3 cells based off the phalloidin-568 staining and normalized to the total number of cells per ×20 field (three to five images were analyzed/treatment condition/experiment). For junctional analyses, bEnd.3 cells were plated onto collagen-coated (Sigma) chambered slides (Thermo Fisher) and grown to confluence. The cells were then treated with vehicle and IFN-γ with or without Y-27632 (ROCKi) for 48 h and fixed with 100% methanol for 10 min. Samples were then incubated with the following primary antibodies: mouse anti-β-catenin (1:100; Cell Signaling), rabbit anti-claudin-5 (1:100; Abcam), mouse anti-pMLC2 (1:100; Cell Signaling), and rabbit anti-VE-cadherin (1:200; Abcam). After incubation with appropriate Alexa Fluor secondary antibodies and DAPI, coverslips were mounted, and the cells were imaged using a Zeiss 780 LSM confocal microscope. ImageJ analysis was used to determine the fluorescence intensity of the junctional proteins and pMLC at the junction and in the cytoplasm by plotting a profile of fluorescence intensity along a 10.375-μm line where the junction was consistently aligned at 4.98 μm. The junctional fluorescence intensity of each protein was considered the intensity at 4.98 μm, and the cytoplasmic intensity was determined as the average of the fluorescence intensity from 0 to 3.735 μm and from 6.225 to 10.375 μm. A depiction of this analysis is shown in Fig. 7D.

### Time-lapse confocal imaging.

Confluent bEnd.3 cells were plated on collagen-coated (Sigma) chambered coverglass slides (Thermo Fisher) and treated with vehicle, IFN-γ, or IFN-γ+Y-27632 (ROCKi) for 24 h. After incubation, the medium was replaced with fresh medium that contained the actin cytoskeleton stain SiR-Actin (100 nM; Cytoskeleton, Inc.) and vehicle, IFN-γ, or IFN-γ+Y-27632 (ROCKi). Time-lapse confocal imaging was performed on a Zeiss 780 LSM confocal microscope with PECON CO_2_ and a temperature-controlled incubation system with CO_2_ set at 5% and 37°C. At least four regions were captured per treatment condition every 3 min for a total of 36 min. These experiments were performed on three separate occasions.

### Mouse brain pericyte cultures, treatments, and immunocytochemistry.

Primary mouse brain vascular pericytes were obtained from iXCells Biotechnologies (10MU-014). All experiments were performed within five passages, wherein cells were grown on collagen-coated (Sigma) chambered slides in pericyte medium containing 2.5% FBS, penicillin-streptomycin, and pericyte growth solution (ScienCell). Once the cells reached ∼70% confluence, they were treated with either DMSO (vehicle) or 100 ng/ml IFN-γ (R&D) for 48 h. Immunocytochemistry was performed by fixing cultures with 4% PFA for 10 min, followed by incubation with the primary antibodies rabbit anti-CC3 (1:100; Cell Signaling), rabbit anti-Pdgfrβ (1:100; Cell Signaling), and rabbit anti-pSTAT1 (1:100; Cell Signaling) for 1 h at room temperature. The samples were incubated with appropriate Alexa Fluor secondary antibody and DAPI and then imaged using a Zeiss 780 LSM confocal microscope. The number of apoptotic pericytes was determined by counting the number of CC3^+^ cells versus the total number of DAPI^+^ cells using Zen software (a minimum of three 20× images were captured and analyzed per experiment; three independent experiments were performed).

### Statistics.

To detect statistically significant differences in the mean values of mock- and T3A-infected mice at one time point (EM analysis), bEnd.3 cultures or pericyte cultures treated with vehicle or IFN-γ (qPCR or immunocytochemistry analysis) and Student *t* tests were used. For analysis that compared more than two groups (e.g., multiple time points or treatment conditions), we used a one-way ANOVA with Tukey’s *post hoc* analysis to detect statistically significant differences between genotypes or treatment conditions. A *P* value of <0.05 was considered statistically significant. Statistics and graphs were all generated using GraphPad Prism 7.03. The standard deviations (SD) are reported on all graphs.

10.1128/mBio.01675-19.1 VIDEO S1 Live imaging of actin cytoskeleton in bEnd.3 cells treated with vehicle. Time-lapse confocal imaging was performed over 36 min of SiR-Actin-labeled actin (white) in bEnd.3 cells after 24 h of vehicle treatment in three separate samples. Scale bar, 50 μm. Download VIDEO S1, MOV file, 5.7 MB. Copyright © 2019 Bonney et al. 2019 Bonney et al. This content is distributed under the terms of the Creative Commons Attribution 4.0 International license.

10.1128/mBio.01675-19.2 VIDEO S2 Live imaging of actin cytoskeleton in bEnd.3 cells treated with IFN-γ. Time-lapse confocal imaging was performed over 36 min of SiR-Actin-labeled actin (white) in bEnd.3 cells after 24 h of IFN-γ treatment in three separate samples. Red arrows point to contracting actin stress fibers. Scale bar, 50 μm. Download VIDEO S2, MOV file, 7.9 MB. Copyright © 2019 Bonney et al. 2019 Bonney et al. This content is distributed under the terms of the Creative Commons Attribution 4.0 International license.

10.1128/mBio.01675-19.3 VIDEO S3 Live imaging of actin cytoskeleton in bEnd.3 cells treated with IFN-γ with ROCK inhibitor. Time-lapse confocal imaging was performed over 36 min of SiR-Actin-labeled actin (white) in bEnd.3 cells after 24 h of IFN-γ+ROCK inhibitor (ROCKi; Y-27632) treatment in three separate samples. Actin stress fibers are present; however, they fail to contract. Scale bar, 50 μm. Download VIDEO S3, MOV file, 5.8 MB. Copyright © 2019 Bonney et al. 2019 Bonney et al. This content is distributed under the terms of the Creative Commons Attribution 4.0 International license.
